# Complete Genome Sequence of Bacillus velezensis GMEKP1, Isolated from a Natural Bamboo Hive of Stingless Bees

**DOI:** 10.1128/MRA.00659-21

**Published:** 2021-11-04

**Authors:** Jaka Widada, Ema Damayanti, Mustofa

**Affiliations:** a Department of Agricultural Microbiology, Faculty of Agriculture, Universitas Gadjah Mada, Yogyakarta, Indonesia; b Research Division for Natural Product Technology, Indonesian Institute of Sciences, Yogyakarta, Indonesia; c Department of Pharmacology and Therapy, Faculty of Medicine, Public Health, and Nursing, Universitas Gadjah Mada, Yogyakarta, Indonesia; University of Maryland School of Medicine

## Abstract

We report the complete genome sequence and annotation of Bacillus velezensis GMEKP1, which was isolated from a hive of stingless bees (Trigona laeviceps). This bacterium has a circular 4,014,839-nucleotide chromosome and two circular plasmids. Genome-mining analysis of the whole-genome sequence revealed that GMEKP1 has 12 biosynthetic gene clusters, dominated by genes encoding polyketide synthase hybrids.

## ANNOUNCEMENT

The stingless bee has been known as a social insect that establishes a symbiotic association with a microbial community (1). Bacillus velezensis GMEKP1 was isolated from a natural bamboo hive of stingless bees (Trigona laeviceps) from Yogyakarta, Indonesia. This bacterium was isolated using a sampling method with slight modification (2). A 5-g hive sample was mixed with 45 ml of sterile distilled water following serial dilution (until x10,000). The isolation procedure used the spread plate method of each serial dilution on International *Streptomyces* Project-2 (ISP-2) medium, with incubation at 30°C for 3 days under aerobic conditions. In another previous study, B. velezensis isolated from stingless bee products of Heterotrigona itama exhibited antimicrobial activity against methicillin-resistant Staphylococcus aureus (3). In this study, we present the annotated genome sequence of B. velezensis GMEKP1 and report the potential active compounds encoded by its biosynthetic gene clusters (BGCs).

High-molecular-weight genomic DNA of the bacterium was isolated using the Nanobind CBB Big DNA kit (Circulomics) from single colonies cultured on ISP-2 medium at 30°C, after 2 days of incubation. The DNA concentration was determined using both a NanoDrop spectrophotometer and a Qubit fluorometer. Library preparation was conducted using kits from Oxford Nanopore Technologies. Nanopore whole-genome sequencing (WGS) data (average read length, 10,114 bp; total number of reads, 329,527) were obtained using GridION sequencing with MinKNOW software version 20.06.9. Base calling was performed using Guppy version 4.0.11 with high-accuracy mode (4). All FASTQ files were filtered using Filtlong software (https://github.com/rrwick/Filtlong), and the quality was visualized using NanoPlot (5). *De novo* assembly was conducted using Flye software version 2.8.1 (6) (37,379 reads [*N*_50_, 13,417 bp]). Medaka software (https://github.com/nanoporetech/medaka) was used to polish the assembled sequence. The assembled contig was aligned to the reference genome using Mauve version 2.4.0 (7). The assembled genomes and their annotation were assessed using both BUSCO version 5.0.0 (8) and CheckM version 0.9.4 (9). The genome completeness was determined to be 99.41%, with 0.0% contamination, and the average sequence coverage was ∼575×. Annotation was added by the NCBI Prokaryotic Genome Annotation Pipeline (PGAP) version 5.2 (best-placed reference protein set; GeneMarkS-2+) (https://www.ncbi.nlm.nih.gov/genome/annotation_prok). Default parameters were used for all software unless otherwise specified.

Full-genome sequencing of strain GMEKP1 led to an assembly of one contig for a total genome size of 4,093,202 Mb, with a GC content of 46.4%. This bacterium has a 4,015,700-nucleotide chromosome and two circular plasmids (13,427 and 64,075 nucleotides). A total of 4,057 genes, 119 pseudogenes, 118 RNA genes, 86 tRNAs, and 5 noncoding RNAs were identified. The average nucleotide identity based on BLAST+ (ANIb) was determined using the JSpecies software tool (10). The ANIb results showed that B. velezensis GMEKP1, isolated from a hive of stingless bees, and B. velezensis strain KACC 18228 (GenBank accession number NZ_LLZA00000000), a rice endophyte (BioSample accession number SAMN04196679), had an ANIb value of 99.97%; this indicates that the two bacteria are the same species, with very high identity.

Genome-mining analysis using antiSMASH version 6.0 (11) revealed that GMEKP1 has 12 regions of BGCs, which encode nonribosomal peptide synthetase modules, polyketide synthase, terpene, bacteriocin, thiopeptide, transacyltransferase polyketide synthase, β-lactone, and other hybrids. These studies provide guidance for further isolation and elucidation studies with antimicrobial compounds of GMEKP1, as well as other biotechnological applications.

### Data availability.

This whole-genome shotgun project has been deposited in DDBJ/ENA/GenBank under accession number CP076450 for the chromosome and accession numbers CP076451 and CP076452 for the plasmids. The BioProject accession number is PRJNA735522, and the BioSample accession number is SAMN19587836. The raw sequence reads are available under SRA accession number SRR16216134. The version described in this paper is CP076450.1.
